# Challenges and opportunities for enhanced cognitive behaviour therapy (CBT-E) in light of COVID-19

**DOI:** 10.1017/S1754470X20000161

**Published:** 2020-05-04

**Authors:** Rebecca Murphy, Simona Calugi, Zafra Cooper, Riccardo Dalle Grave

**Affiliations:** 1 The Centre for Research on Eating Disorders at Oxford (CREDO), Department of Psychiatry, University of Oxford, Oxford OX3 7JX, UK; 2 Department of Eating and Weight Disorders, Villa Garda Hospital, Via Monte Baldo, 89, 37016 Garda (Verona), Italy; 3 Department of Psychiatry, Yale School of Medicine, 157 Church Street, New Haven, CT 06510, USA

**Keywords:** anorexia nervosa, binge-eating disorder, bulimia nervosa, cognitive behaviour therapy, coronavirus, COVID-19, distance therapy, eating disorders

## Abstract

In the past few weeks, coronavirus disease 2019 (COVID-19) has dramatically expanded across the world. To limit the spread of COVID-19 and its negative consequences, many countries have applied strict social distancing rules. In this dramatic situation, people with eating disorders are at risk of their disorder becoming more severe or relapsing. The risk comes from multiple sources including fears of infection and the effects of social isolation, as well as the limited availability of adequate psychological and psychiatric treatments. A potential practical solution to address some of these problems is to deliver enhanced cognitive behaviour therapy (CBT-E), an evidence-based treatment for all eating disorders, remotely. In this guidance we address three main topics. First, we suggest that CBT-E is suitable for remote delivery and we consider the challenges and advantages of delivering it in this way. Second, we discuss new problems that patients with eating disorders may face in this period. We also highlight potential opportunities for adapting some aspects of CBT-E to address them. Finally, we provide guidelines about how to adapt the various stages, strategies and procedures of CBT-E for teletherapy use in the particular circumstances of COVID-19.

**Key learning aims:**

(1) To appreciate that CBT-E is suitable for remote delivery, and to consider the main challenges and potential advantages of this way of working.
(2) To identify and discuss the additional eating disorder-related problems that may arise as a result of COVID-19, as well as potential opportunities for adapting some aspects of CBT-E to address them.
(3) To learn how to adapt CBT-E for remote delivery to address the consequences of COVID-19. Specifically, to consider adaptations to the assessment and preparation phase, the four stages of treatment and its use with underweight patients and adolescents.

## Introduction

Coronavirus disease 2019 (COVID-19) has dramatically expanded with a major outbreak across the world (Paules *et al*., 2020). To limit the number of COVID-19 cases and deaths, most governments around the world have decided to put their countries on lockdown. ‘Lockdown’ takes various forms but usually means that restaurants, bars, shops, schools and gyms are closed, and people are required or asked to stay home. Other related practices during this time include ‘social distancing’ (keeping physical space from other people outside of your household), ‘quarantine’ (keeping someone who might have been exposed to COVID-19 away from others) and ‘isolation’ (keeping people with confirmed cases of disease separated from people who are not sick).

In this difficult situation, individuals with eating disorders may be at a high risk of relapsing or of a worsening of the severity of their disorder. Aside from the particular problems raised by increased fears of infection, social distancing and self-isolation, psychological and psychiatric services have become less available than usual. This situation urgently requires a new way to deliver psychological treatments, given that face-to-face treatment is not viable in these circumstances. Standard procedures for addressing eating disorder psychopathology also require modification in light of the additional problems caused by COVID-19.

A potential practical solution to address some of these problems is to deliver enhanced cognitive behaviour therapy (CBT-E: Fairburn *et al*., 2003; Fairburn, 2008) remotely. The most recent NICE guidelines (National Institute for Health and Care and Clinical Excellence, 2017) recommend CBT-ED as an evidence-based treatment for all eating disorders. CBT-E is one major example of the specialist cognitive behavioural therapies for eating disorders covered by the umbrella term ‘CBT-ED’. Many of the CBT-E interventions described below are also used in other models of CBT for eating disorders (CBT-ED).

In this guidance we address three main topics.

(1) The possibility of delivering CBT-E remotely;
(2) New problems to be addressed and potential opportunities as a result of COVID-19;
(3) Adapting CBT-E for distance use, with particular emphasis on its use in the light of COVID-19.

Clearly there are many challenges to therapists and patients who find themselves suddenly having to work differently and deal with new problems (Beat, 2020; Dalle Grave, 2020). However, it is also worth considering any potential opportunities that the current circumstances present.

This guidance will not cover security, regulatory and generic risk requirements of distance therapy as this is covered elsewhere (e.g. need to update security regularly on devices, password protection, encryption, secure storage of information, informed consent, need to obtain appropriate information to manage risk) (NHS, 2020), and it will not advocate any particular technology platforms. Each therapist will need to consider these carefully before embarking on distance CBT-E (e.g. in the USA, there are specific telehealth practice guidelines and insurance considerations) (American Psychological Association, 2013). The BABCP has provided useful guidance for psychological professionals during COVID-19 (see ‘Further reading’ section).

## Delivering distance CBT-E

### CBT-E is suitable for use remotely

CBT-E is well suited to many aspects of distance therapy. It is a talking therapy and thus this aspect can be relatively easily retained through online video-calls. CBT-E emphasises the importance of the patient making changes and working on specific tasks between sessions to bring about this change (Fairburn *et al*., 2008a); this is perfectly consistent with working remotely. Indeed, with distance therapy, the patient may be less likely to attribute progress to the therapist and more likely to have an improved sense of self-efficacy (Vallejo *et al*., 2015).

Despite an absence of data on delivering distance CBT-E, in our view it should still be possible to develop a good collaborative therapeutic relationship and deliver most aspects of treatment. In our experience, many of the challenges of this form of delivery can be relatively easily overcome when working with patients with whom the clinician has already engaged during initial face-to-face treatment. More challenging is delivering treatment entirely remotely without any initial face-to-face contact, as it might be more difficult to develop a collaborative relationship. However, the non-verbal communication aspects of the therapeutic relationship (i.e. facial expression, eyes, voice, body posture and gestures, physical appearance) are only slightly compromised by online treatment provided video is possible (Glueck, 2013). Indeed, a study of remote delivery of psychological treatment for depression and generalised anxiety found that a high level of therapeutic alliance could still be maintained (Hadjistavropoulos *et al*., 2017). There is also evidence to suggest that a positive therapeutic alliance is driven by early improvement (Graves *et al*., 2017). As such, CBT-E’s goal to produce an early reduction in eating disorder features may help to establish a positive therapeutic relationship even in remote delivery.

With regard to outcome, a study of cognitive behavioural therapy for bulimia nervosa delivered via telemedicine was found to be acceptable to patients and to produce similar outcomes to face-to-face delivery (Mitchell *et al*., 2008). Research on delivering other psychological treatments through video calls, although somewhat limited, suggests that assessments and therapy sessions can work well using this mode of delivery (Backhouse *et al*., 2012; Hulsbosch *et al*., 2017; O’Reilly *et al*., 2007; Stubbings *et al*., 2013). Therefore, we remain hopeful about this option.

While there are additional issues around maintaining confidentiality and working securely, we believe these issues can be overcome with guidance and experience.

### Mode of delivering distance CBT-E

Delivering CBT-E remotely is possible using a variety of video-call platforms (e.g. Zoom, Skype). The choice will depend on various factors, including: the experience, availability and preferences of the patient and therapist; any constraints of services and regulations such as confidentiality compliance; and how well the platform functions with limited bandwidth or over-demand (Owings-Fonner, 2019). If there is more than one option available, it is worth considering specific features offered by each platform. For example, with some platforms (e.g. Zoom) it is possible to screen share and choose the ‘whiteboard’ option where it is possible to draw in real-time.

We suggest keeping sessions to approximately the same length as face-to-face (possibly with a break in the middle if a continuous session is not feasible).

Audio calls are likely to be less satisfactory in terms of fidelity to CBT-E and the therapeutic alliance, as it will not be possible to share materials live onscreen or observe some important aspects of non-verbal communication. Given the potential inequalities in digital provision, it is important to be flexible about what is possible. In some cases, audio calls may be the only option, and probably something is better than nothing. It may be easier if the therapist and patient have already met face-to-face, but again this may not always be possible. It may be possible to send any documents (via email or other methods, taking into account issues of security and confidentiality, while in some cases this may not be permissible). If documents cannot be shared, the patient could take the therapist through them verbally (with some helpful and sensitive guidance from the therapist about how to do this in a timely manner).

As an alternative to CBT-E, the telephone can be used to deliver guidance to accompany the printed self-help version of CBT-E, *Overcoming Binge Eating* (Fairburn, 2013). This method of delivering telephone guidance to accompany self-help in bulimia nervosa and related disorders has been found to work well (e.g. Palmer *et al*., 2002). However, while this option may be suitable for some patients, its use with underweight patients is not supported.

### Challenges of distance CBT-E

Distance therapy may produce particular challenges, in particular for those who are not already familiar with technology and who have little privacy at home, or those who remain unconvinced that this mode of therapy will work. In addition, for many patients with eating disorders, seeing themselves on video calls may result in their focusing more on their appearance, thus providing a further opportunity for in-session body checking (see chapter 5, *CBT and Eating Disorders*). In this circumstance, the therapist could spend time reviewing the user instructions for different platforms, pointing out, for example, that with some platforms (e.g. Zoom) the patient can choose to ‘hide myself’ whilst still being seen by the therapist. On the other hand, video calls can be an opportunity for the patient to build tolerance to viewing themselves and practise ways of not checking their appearance in unhelpful ways (e.g. scrutinising specific parts of the body). Building such tolerance may be especially important during social distancing, as otherwise this could create problems for the patient in other forms of virtual socialising. Therapists can encourage patients to become used to seeing themselves on video as an experiment to help practise refocusing their attention on other things such as looking at the background environment or at a more neutral part of the body (e.g. hair, hands).

### Advantages of distance CBT-E

Distance therapy has clear advantages too. For patients it is often more convenient, causes less disruption to life and may be less costly. It also occurs in the same place as patients spend their time, potentially avoiding a context-dependent change only. It may also mean that twice-weekly sessions (where appropriate) are easier to implement.

It is helpful for therapists to share with the patient that CBT-E is well suited to many aspects of working online and to be hopeful about this way of working. However, the therapist may need to be more mindful than usual of signs of loss of motivation on the part of the patient. Engagement is likely to be more of a priority in this mode of working.

## New problems and potential opportunities arising from COVID-19

Some inevitable consequences of COVID-19, in particular the prolonged social isolation (Brooks *et al*., 2020) and the fear associated with possible infection, may interact negatively with eating disorder psychopathology and the provision of CBT-E treatment (Dalle Grave, 2020). If patients do not live alone, then spending increased time with household members may bring additional strains. However, these problems may also become potential opportunities for improving the outcome of treatment. Some patients find that spending more time with loved ones at home is supportive and helpful while having CBT-E.

### Making treatment a priority

Some patients may struggle with focusing on the eating problem in therapy sessions and may want to spend time talking about COVID-19. Patients who are seeking or receiving treatment might feel guilty or not deserving of treatment, especially in light of the media focus on COVID-19. They may find it even harder to share concerns about their eating, shape and weight in case they seem trivial.

More positively, for those patients who have a reduction in workload or social commitments, this may be an opportunity to focus their efforts on treatment with fewer competing demands. This fits with CBT-E’s stance that the more one puts into treatment the more one will get out of it (Fairburn *et al*., 2008a). As therapists and patients may be taking fewer breaks or holidays, this may lead to fewer interruptions of treatment and help to maintain therapeutic momentum.

### Over-evaluation of shape and weight

**Figure 1.**
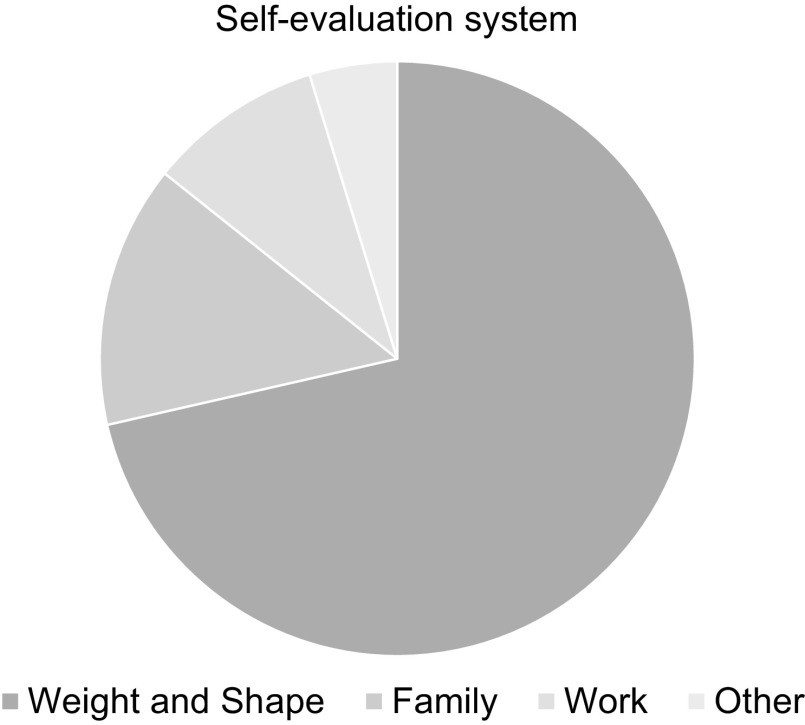
A system for self-evaluation dominated by shape and weight.

CBT-E considers the ‘core psychopathology’ of eating disorders to be the over-evaluation of shape and weight and their control; that is, the judging of self-worth largely, or even exclusively, in terms of shape and weight and the ability to control them, as shown in Fig. 1 (see Fairburn, 2008; chapter 8). In many cases the desire to control one’s eating, weight and shape may be intensified during a stressful period such as the present circumstances where other aspects of life feel beyond one’s control (Fairburn *et al*., 1999). The need for shape, weight and eating control may also be intensified by the fear and anxiety associated with the risk of being infected by SARS-CoV-2. Although, on the other hand, some patients report that the precautions advised to prevent infection help them to distract themselves from their concerns about eating, shape and weight.

Patients’ narrow focus on eating, shape and weight as a way of evaluating themselves may not be helped by the newly increased narrowing of life and opportunities imposed by the current restrictions to slow the pandemic. It will probably be more difficult to expand and develop new domains for self-evaluation. Pre-occupation with what one has eaten and body concerns may dominate without other competing interests and activities being so easily available. It is also difficult to address some expressions of over-evaluation (e.g. some forms of body avoidance) while other expressions may be intensified (e.g. body checking and labelling negative emotions as feeling fat). Social isolation may mean that people have reduced opportunities to interact with people without eating disorders and an increased use of social media may in fact exacerbate concerns. For example, patients may spend more time viewing online material concerned with eating, shape and weight. In addition, with virtual socialising, patients may feel even more judged by their on-screen appearance and may start to become pre-occupied with their video images.

More positively, social media may be more commonly used as a means of socialisation at the current time. This may help individuals with eating disorders feel connected or a part of a community. Indeed, some patients may be less concerned with their appearance and eating given the focus on COVID-19 and reduced socialising.

### Strict dieting

For patients who are dieting strictly, there is a challenge that particular diet foods may be more difficult to obtain. If patients are trying to regain weight, foods that they have planned to eat may not be available. It is difficult or impossible to address some dietary rules, such as social eating and eating in restaurants.

More positively, it is an opportunity for patients to be more flexible with their eating (in the same way that holidays in different countries also present such opportunities). Often patients describe being ‘too busy to eat’ and finding it hard to fit regular eating within their schedules, but this is less likely to be the case during this time. However, if patients are frontline workers they may in fact be much busier than usual, and it may be harder for them to find time to eat regularly or even to buy food.

### Binge eating

For patients who binge eat, there are additional challenges that may trigger dysregulated and excessive eating and binge-eating episodes: being around food at home all day; the difficulties in obtaining ‘safe foods’; the need to purchase ‘in bulk’ online or during limited shopping trips rather than shop more frequently; the lack of the helpful structure of their usual routine of work; an increase in stressful life events and adverse moods (e.g. strained relationships due to spending more time with members of one’s household or being alone); and fewer options for distractions. More positively, buying some binge foods that are usually eaten during binge-eating episodes may be more difficult.

### Exercise

For patients who exercise for shape/weight reasons, their fear of weight gain may result in their finding it harder to eat given the restricted opportunities for exercise and ‘burning calories’. Patients who exercise to modulate mood are likely to have increased difficulties and may increase dieting or binge eating instead. It may also be that patients engage in more individual forms of exercise instead of, arguably, ‘more healthy’ social exercise. Some patients may, however, find that the closure of gyms and health clubs offers some helpful respite.

### Low weight

Underweight patients may be at higher risk of medical complications associated with malnutrition as they may have more limited access to medical treatments. This emphasises the importance of not delaying treatment and weight regain so as to be as healthy as possible. The current circumstances (e.g. staying at home, reduced socialising) may mean that it is harder for underweight patients to recognise the impairment and the restrictions to life that the eating disorder is causing.

### Co-morbidities

For patients who have eating disorders co-morbid with other conditions (such as depressive disorders, anxiety disorders, obsessive compulsive disorder, post-traumatic stress disorder, and substance use disorder) the rumination, pre-occupation and anxiety triggered by the COVID-19 pandemic can accentuate the severity of their condition. In turn, this worsening of the co-morbid condition is likely to interact negatively with eating disorder psychopathology.

Patients with co-morbid severe obesity appear to be at more risk of negative health consequences of COVID-19 (Kassir, 2020; Simonnet *et al*., 2020). This may mean that some patients are more anxious during this time. However, regular eating and reducing binge eating remain helpful goals for which to aim.

## Adapting CBT-E for distance delivery in light of COVID-19

### Assessment and preparation phase

#### Suitability for distance CBT-E

In some cases, it may be possible to consider postponing therapy until it is possible to deliver CBT-E face-to-face. Often this will not be possible or will create more problems for patients, services and therapists later. If patients and therapists are available and wish to make treatment a priority, if there are no other obstacles to treatment (e.g. clinical depression, daily substance misuse, difficulties using online technology or participating in the planned online sessions, competing commitments), and if the therapist does not have concerns about his/her competence in delivering CBT-E online, we suggest that treatment should go ahead. This mode of working requires that the patient and therapist are able to meet privately and securely (e.g. in a closed room).

We suggest that if patients present with high physical risk [e.g. due to a severely low body mass index (BMI), i.e. <16.0; or rapid weight loss of 1 kg/week; frequent purging behaviour] then a separate medical assessment is needed to determine if distance therapy is appropriate. Such an assessment should also consider whether there is a need for ongoing (regular or occasional) in-clinic weighing. In other cases, for patients who are not at risk, as weight cannot be measured in the therapist’s office, the remote collaborative weight procedure described later in Stage One can be used for assessing weight.

If a patient is at high risk of immediate harm to themselves, then this mode of working is unlikely to be suitable. In all cases, it is important during the assessment phase to discuss a crisis or safety plan, including having a system in place for local crisis intervention with appropriate contact details.

In CBT-E, a standardised measure of eating disorder psychopathology, the EDE-Q (Fairburn and Beglin, 2008), and a measure of eating disorder related impairment, the CIA (Bohn and Fairburn, 2008) are recommended during the initial assessment and to measure progress later in treatment. Although these have not been evaluated for online use, it may be possible for clinicians to use them remotely. The EDE-Q and CIA are under copyright but no permission is required for non-commercial clinical or research use. They could be administered using an online survey platform (e.g. Microsoft Forms), with appropriate author acknowledgements and the use of the same wording and response options. Alternative options include electronically sending the measures to patients for them to print and return electronically once completed.

#### Engaging the patient in treatment and change

In face-to-face CBT-E, engaging the patient is the top priority (see chapter 5, Fairburn *et al*., 2008a). This is also true for distance CBT-E and similar strategies should be used. However, more effort should be placed on involving the patient in the assessment, encouraging the patient to speak openly and to ask questions. It is advisable to ask patients how they feel about having treatment online. The therapist could explain: ‘We do not know if delivering CBT-E remotely has any different effects from doing so face-to-face. However, we know that the therapy has the best chance of success if you carry out the “next steps” that we agree on for you to work on between our sessions. We feel hopeful that if we work together to deal with any challenges arising from distance therapy that it has a good chance of being successful’.

#### Assessing the nature and the severity of the psychopathology present

In CBT-E, the assessment conducted by the therapist at the outset of therapy covers a broad range of topics. It is designed to obtain information needed to formulate an individual patient’s eating disorder (see Table 5.1 in chapter 5, Fairburn *et al*., 2008a). The assessment of the eating problem does not require any modification compared with face-to-face CBT-E.

#### Explaining what treatment will involve

In CBT-E, a key aspect of the first stage of treatment is to explain to the patient what treatment will involve, with various topics being covered (see Table 4.2, chapter 4, Fairburn, 2008). This requires only minimal modification compared with face-to-face CBT-E. In addition to enquiring about the patient’s attitude to treatment, we suggest that the therapist specifically asks about the patient’s attitude to receiving therapy remotely. In terms of describing the practical aspect of treatment, time should also be dedicated to discussing how the online sessions will be conducted. This can include contingency plans for loss of internet connection. It is crucial to establish and share with the patient what will be required to implement online CBT-E. This will include asking them to have sessions in a closed room without interruptions (American Psychological Association, 2013).

**Figure 2.**
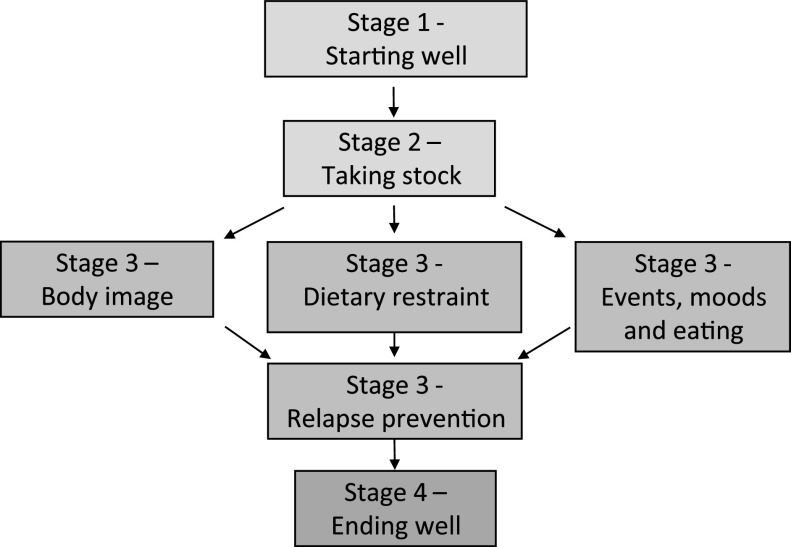
CBT-E treatment map.

CBT-E has four stages (see Fig. 2) and adaptations to these for remote delivery during COVID-19 are described below.

### Stage 1

It is crucial that treatment gets off to a good start and this stage is described in the therapist’s guide for CBT-E (see chapters 5 and 6, Fairburn, 2008).

#### Setting and following an agenda

In CBT-E, each session starts with a brief review of how things have been going since the last session and collaboratively setting an agenda. If treatment has already begun face-to-face, and there is a transition to distance therapy, the topic of having online treatment should also be added to the agenda. This discussion can include enquiring about the patient’s attitude towards online treatment.

It may be that patients wish to spend the session talking about COVID-19 worries and consequences. As is the case in standard CBT-E, it may be possible to relate these other issues directly to the therapeutic procedures being covered in this stage of treatment. For example, when planning meals, any changes in food availability may be discussed as part of regular eating, or problem-solving can be used to address day-to-day difficulties which arise from the COVID-19 pandemic. The therapist can validate the patient’s natural concerns surrounding COVID-19 and its consequences whilst at the same time explaining that it is crucial to focus the therapy on overcoming the eating disorder. A case can be made that, if patients make progress in overcoming their eating problem, they are in a better position to deal with the difficulties of the current situation.

#### Jointly creating the formulation

The creation of a personalised visual representation of the processes that appear to be maintaining the patient’s eating problem is a crucial aspect of CBT-E. In face-to-face CBT-E the therapist draws the diagram on paper, building it up step-by-step in collaboration with the patient. In order to still draw up the formulation in a collaborative manner in distance CBT-E, it is helpful for the therapist to regularly share the diagram with the patient as it is being created. This might be achieved by regularly presenting it to the camera as it is created and then the final image can be scanned in and shared via screen share or something similar. If the video call platform allows (e.g. Zoom) a whiteboard function can enable live drawing in the session.

#### Confirming homework assignments, summarising the session and arranging the next appointment

The patient’s implementation of ‘homework’ is central to CBT-E, although the term ‘Next Steps’ is used with patients to avoid any unhelpful connotations. At the end of the session, the main take-home messages are summarised and the next appointment is arranged. These aspects of CBT-E do not require any modification compared with face-to-face CBT-E.

#### Establishing real-time self-monitoring

Real-time self-monitoring is the ongoing ‘in-the-moment’ recording of relevant behaviour, thoughts, feelings and events. It is initiated from the outset of CBT-E, continues throughout most of the treatment and is central to it. Usually therapists give their patients several paper records to complete by hand (these are available on: www.cbte.co) and patients bring their completed records to the next session.

**Figure 3.**
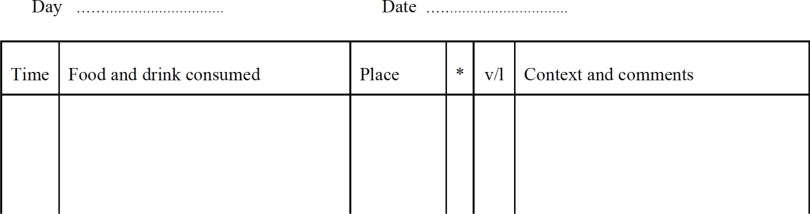
Self-monitoring record.

There are various options which can be considered in distance CBT-E:

- For those patients who have a printer at home, the therapist can email the patient a copy so that self-monitoring can be carried out using paper and pen.
- For those who do not have a printer at home, this is more difficult. It may be possible to post copies to patients (if permitted). If this is not feasible, the patient can simply create their own monitoring records at home on paper or in a journal or diary customised to reflect the columns used in CBT-E (see Fig. 3).
- Alternatively, word-type packages on a smartphone device or similar could be used for patients who are familiar with this technology e.g. ‘notes’ or ‘word’ or similar.

Patients should be advised to create the same columns as in face-to-face CBT-E if creating their own records.

#### Reviewing the recording

In CBT-E, the therapist reviews each self-monitoring record in turn at the start of every session. In distance CBT-E, ideally patients should send the scanned/photographed/e-copies of monitoring records in advance of the session to the therapist (e.g. by email – considering security and confidentiality issues). Then reviewing the recording can follow the same procedure as in face-to-face CBT-E. If this method is not possible, then another option could be for the patient to be guided to take the therapist briefly through the records at the start of the session.

#### Collaborative weekly weighing

Given that fewer than 40% of clinicians report weighing patients (Cowdrey and Waller, 2015), the barriers posed by the COVID-19 pandemic probably risks the omission of regular weighing from virtual sessions. However, we strongly recommend retaining the collaborative in-session weighing, including reviewing the weight graph together, as this is a crucial aspect of CBT-E (Fairburn *et al*., 2008a).

It may be possible for the patient to move their video-call device so that the therapist can be present for the actual weighing and respond in a manner as close to standard treatment as possible (e.g. ‘Now it is time for weekly weighing. If you can stand on the scales and read out the number. Okay so that is X. Let’s review this together in our session’) or the therapist can prompt the patient to do this whilst waiting.

We suggest that the therapist is responsible for preparing and keeping the weight graph, as usual. Screen sharing can be used if the system allows (by scanning in the pen-and-paper document or using an electronic graph) or the therapist can hold up the graph to the camera during the video call.

In usual CBT-E practice there is careful preparation before patients use their own weighing scales towards the end of treatment and this includes a period of recalibration where the therapist’s reading is compared with the patient reading (see Fairburn et al., 2008a). As this is not possible, it can be reframed as an opportunity to be more flexible about the error surrounding each reading. As, of necessity, the readings are from a different set of scales, this can be indicated on the graph (e.g. by drawing a line or arrow to show when this change occurred). It will obviously not be possible to draw conclusions initially on the basis of the difference between the two readings. However, once the new scales are used regularly, change can be interpreted in the usual way when sufficient readings have been obtained.

We recommend that therapists follow usual CBT-E guidance for advising patients on purchasing and using scales (e.g. keeping scales out of reach in between weighing times for patients who are at risk of frequent weighing), although in standard practice this only becomes relevant towards the end of treatment. In standard CBT-E, the handover of weighing comes later in treatment when the eating disorder mindset is no longer in place. However, in the case of distance CBT-E, the therapist will need to discuss with patients the importance of giving honest reports on the number on the scales.

#### Educating the patient about eating problems

In CBT-E, ‘guided reading’ is used to help educate patients about eating problems. This does not require any modification when delivered remotely. The use of guided reading *Overcoming Binge Eating* (2nd edition; Fairburn, 2013) should be encouraged.

#### Establishing regular eating

The ‘regular eating’ intervention is considered to be the foundation upon which other changes in CBT-E are built. It involves patients eating planned meals and snacks at regular intervals whilst using strategies to resist urges to eat in the gaps. Implementing this intervention requires little modification compared with face-to-face delivery. In terms of the positive features of regular eating, as noted earlier, patients may have more time to plan their eating and to make sure they eat regularly. However, it may be harder to purchase acceptable foods, and those working longer hours and/or in keyworker roles (e.g. healthcare personnel) may encounter difficulties eating regularly.

In terms of sticking to a regular eating plan and not eating between regular meals and snacks, patients may find it helpful to plan their other activities in addition to their eating plan. This can be helpful in standard CBT-E for patients who have lives that are lacking in structure and may be especially relevant during this time. Planning times for meals and snacks can help to create some structure and patients can include activities for the time between these meals. Patients can be encouraged to try to avoid spending too much time in the kitchen area, although this may be more difficult and so may require particular effort. If patients are at risk of binge eating whilst cooking, it may be helpful for them to cook meals at safe times of the day and portion these (e.g. the morning) in preparation for other, more risky times. Alternative activities may be more constrained, and may not be social, as suggested by CBT-E (Fairburn *et al*., 2008a), but the therapist can still help the patient to develop a list of engaging activities that are not compatible with eating.

#### Addressing the patient’s style of eating, purging, feeling fullness

In CBT-E, patients’ ‘eating style’ is addressed if there are obvious problems or if there is a tendency to over-eat. Non-compensatory purging often needs to be identified and addressed, whereas purging, which serves a compensatory function, will often dissipate when patients regain control over their eating. If feeling full or responses to feeling full are a barrier to overcoming the eating problem, they also need to be addressed. The procedures used to address these features do not require any modification when delivered remotely, although they may be more important during this time (e.g. the need to eat in a set place or coping with fullness when there are fewer distractions).

#### Addressing excessive exercising

Excessive exercising is most commonly seen in underweight patients. If it is undertaken for weight control, it generally decreases during the course of CBT-E as patients become less concerned about their eating, shape and weight. It presents a particular problem in inpatient settings where physical activity is more limited, and patients often feel ‘cooped up’. During COVID-19, the limited possibility of walking and exercising may increase the fear of weight gain. This fear of weight gain should be addressed with standard CBT-E procedures (Fairburn *et al*., 2008a).

More positively, isolation at home gives patients the opportunity to change unhelpful and excessive exercising. Healthy and planned exercise that can be carried out at home should be discussed and encouraged. It may even be possible for patients to engage in virtual social exercise or to ask parents or siblings to exercise with them.

#### Involving significant others

CBT-E’s practice of involving significant others if they can help the patient to make changes or if they are making it difficult for the patient to make changes (see Fairburn *et al*., 2008a) can be implemented as usual. Involving significant others may be especially helpful in an attempt to create an optimum home environment during a prolonged period of social distancing. Close and prolonged contact with family members may trigger or accentuate interpersonal difficulties and associated mood changes that might contribute to the maintenance of eating disorder psychopathology (e.g. increasing dietary restraint and/or the number of binge-eating episodes) (Fairburn *et al*., 2003). Additionally, meetings with significant others offer an important opportunity to provide families with support at a time when the challenges of living with someone with an eating disorder may be more apparent. Sessions with significant others should take place just as in standard CBT-E, i.e. immediately after a routine session (Fairburn *et al*., 2008a).

### Stage 2

This is a transitional stage that involves a joint review of progress and the design of Stage 3 (see chapter 7, Fairburn, 2008). Distance CBT-E can make use of the same Stage 2 strategies and procedures. The identification of any existing barriers to change should also include the evaluation of patients’ attitudes towards distance treatment, the use of online CBT-E procedures, and any problems associated with isolation or fear of infection.

### Stage 3

This is the main body of treatment during which the therapist addresses the key mechanisms that are maintaining the patient’s eating disorder (e.g. ‘over-evaluation of shape and weight’). The implementation of Stage 3 varies from patient to patient, and is described in the treatment guide (see chapters 8–10, Fairburn 2008).

If concerns about COVID-19 have intensified the patient’s desire to control eating, shape and weight, the therapist could explain that it is often helpful to have treatment in the face of difficulties, whilst appreciating that this may make change more difficult. The therapist can help the patient to think of more helpful ways of coping.

#### Body image module

##### Identifying the over-evaluation and its consequences

In distance CBT-E, the same procedures can be used. This module begins with identifying the patient’s scheme for self-evaluation and drawing a pie chart to represent this system (see Fig. 1 for an example of a typical patient’s pie chart). When working remotely, so as to draw the first version of the pie chart in a collaborative manner, it is helpful for the therapist and patient to regularly share the diagram as it is created – perhaps by showing this to the camera. The final image could perhaps be scanned and shared via screen-share.

**Figure 4.**
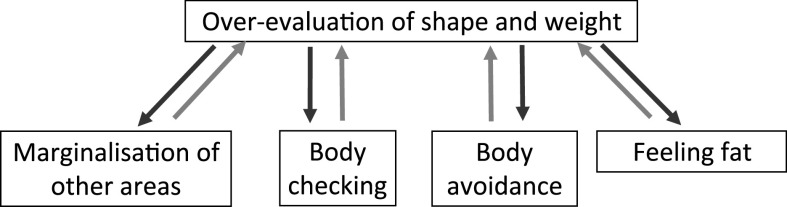
Extended formulation.

This step is followed by a plan for addressing the over-valuation of shape and weight, using the strategies described in Fig. 4.

##### Enhancing the importance of other domains for self-evaluation

The goal of this aspect of treatment is for patients to begin to engage in other marginalised areas of life, and for these areas to become more important in the patient’s system for self-evaluation. The marginalised areas tend to include relationships with others and as such interpersonal activities are encouraged. Given this, isolation presents an important obstacle. However, patients can be encouraged to consider what their friends or work colleagues do in their time of social distancing. Therapists will need to collaborate with patients to think creatively about what can be done to develop other areas despite the constraints (e.g. developing hobbies and interests that can be carried out at home or in the garden if available, or those which can be developed online and in preparation for the future).

##### Addressing shape checking and comparison making

Shape checking tends to be especially important in maintaining concern with body shape. Under many circumstances, body checking monitoring can be used and reviewed as in standard CBT-E (Fairburn *et al*., 2008a) provided that the technology allows. Similar procedures are used to address shape checking behaviour that should be stopped, and mirror checking that may need to be adjusted.

Isolation at home is likely to reduce the opportunity for comparison checking with other people in the real world (perhaps with the exception of household members, e.g. siblings), but it might increase comparisons with images in the media, in social networks and during online meetings. It is important that online activity is included when patients monitor comparison-making. Some homework tasks to address comparison-making (e.g. selecting every third person and scrutinizing other people’s bodies) are difficult to implement during isolation at home. However, patients may be asked to research online examples of body image manipulation (airbrushing and similar) and provided with education about this.

##### Addressing shape avoidance

Shape avoidance, such as avoiding looking in the mirror, also needs addressing as it allows concerns and fears about shape and appearance to persist without information about one’s actual appearance. Isolation is not an obstacle to encouraging patients to get used to the sight and feel of their body. However, it can be more difficult to enable patients to get used to others seeing their body and to be involved in some activities that involve a degree of body exposure. Activities that can be easily done at home include applying body lotion, having a massage (self-massage or by someone in one’s household) and yoga.

##### Feeling fat

CBT-E conceptualises feeling fat as the result of the mislabelling of certain emotions and bodily experiences (for example feeling hot, lethargic, bored or sad). This experience may be increased, in the current circumstances, for patients who are experiencing a reduction in activity and productivity and who associate this with feeling fat. This should be addressed using standard CBT-E procedures.

#### Dietary restraint module

For patients with strict dietary rules, CBT-E encourages flexibility (see Fairburn *et al*., 2008a). The current situation may be an opportunity to reinforce the view that it is helpful to have guidelines rather than rules about one’s eating. There may be restrictions on the types of foods available and patients’ usual options may be unobtainable. Patients can be encouraged to use recipes or meal plans as a guideline but recognise that items will often need to be substituted. For those patients who are underweight, they can be encouraged to try to substitute for foods with similar energy values (not exactly the same!). Although it is not advisable for patients to spend too much time looking at recipes, it is possible to Google ‘coronavirus recipes’ to get ideas for various substitutions.

To avoid patients eating alone, if there are no others to join them in their household, they can perhaps try virtual social meals.

Making a list of avoided foods by visiting the supermarket is not advised. Even online shopping may not be possible. Alternatives include looking at online lists of foods (e.g. Wikipedia has one). Purchasing a wide range of avoided foods may be more difficult but patients should still be able to obtain a sufficient number of avoided foods to address their concerns and practise flexibility.

For patients who binge eat and who also need to buy in bulk, it can be helpful (where possible) to choose frozen foods as these will be less accessible during an urge to binge. Patients could also be encouraged to store extra food supplies carefully, possibly in less accessible places, or to portion and store foods in smaller quantities.

#### Events, moods and eating module

If in Stage 3 events and moods appear to be contributing to the maintenance of the eating disorder, this probably needs to be addressed. Examples of the ways in which this can occur includes patients eating less to gain a sense of control when outside events seem beyond their control, or using binge eating or purging to cope with difficult events or moods. This module might be used more often than in standard CBT-E, as prolonged quarantine is often associated with negative psychological effects including post-traumatic stress symptoms, confusion, boredom and anger (Brooks *et al*., 2020). All these changes in mood may negatively influence patients’ eating and should be addressed using standard CBT-E procedures.

#### Setbacks and mindsets module

The core psychopathology of eating disorders can be viewed as a ‘mindset’. In CBT-E, patients learn about mindsets and how to control them. The same strategies and procedures used to achieve this during face-to-face work can be employed when delivering CBT-E remotely.

#### Broad CBT-E modules

The broad form of CBT-E is designed for patients in whom mechanisms external to the eating disorder psychopathology are also contributing to the maintenance of the eating disorder and preventing change. Additional ‘modules’ are used to address these external mechanisms (see chapter 13, Fairburn, 2008). The interpersonal difficulties module may be considered and used more often as interpersonal difficulties may arise or be accentuated due to prolonged social distancing or quarantine (e.g. isolation from friends, disputes with family members) and these might serve to maintain the eating disorder.

The other external maintaining mechanisms should be addressed as with standard CBT-E procedures only if they are severe, seem to contribute to the maintenance of the eating disorder, and create an obstacle to treatment (Fairburn *et al*., 2008b).

### Stage 4

This is the final stage in treatment and focuses on ending treatment well. It is possible to use the usual strategies and procedures when delivering this intervention remotely (see chapter 12, Fairburn, 2008). However, time should also be dedicated to evaluating the strategies that the patient could adopt at the end of quarantine or social distancing. This is important in order to help patients consider (i) ways in which to make further progress when there is freedom from lockdown and (ii) how to deal with high-risk situations such as eating with others or social situations which could trigger concerns about shape and weight.

## Post-treatment review sessions

In this difficult period, it is advisable, if it is possible, to plan more frequent post-review sessions than is usual practice (e.g. after 4, 12 and 24 weeks) to support patients and to identify and promptly address early signs of relapse.

## Adaptations for underweight patients

CBT-E includes an additional module for underweight patients and those who under-eat (see chapter 11, Fairburn, 2008). As noted, distance CBT-E is not indicated for patients with a BMI <16 and in those who have a weight loss greater than 1 kg per week over the last 8 weeks.

If possible, underweight patients should be assessed by a physician to assess if their physical state is compatible with distance treatment and to manage any medical complications if indicated. Clinicians should be flexible in taking the decision to treat underweight patients with distance CBT-E, as many inpatient treatments have stopped admitting patients and distance treatment may be the only treatment available for many. In addition, it is important to consider the consequence of not providing any treatment over this time.

Stage 1 of distance CBT-E uses the same adaptations described above for non-underweight patients, and those described in the CBT-E guide for underweight patients (Fairburn *et al*., 2008a). However, in discussing the pros and cons of change and addressing weight gain, therapists should encourage patients to consider the importance of restoring a low normal weight to overcome the medical complications associated with malnutrition that may increase physical risk in the case of COVID-19 infection.

In Stage 3, the therapist should consider if necessary giving more advice than standard CBT-E on how to plan meals and snacks with the aim of creating a 500 kcal energy-positive balance. However, the therapist should always be aware that there is a danger that patients may transform this guidance into a dietary rule and should take steps to guard against this.

## Adaptations for adolescent patients

Therapists should refer to the new CBT-E manual for adapting CBT-E for adolescents (Dalle Grave and Calugi, 2020). Usually, adolescents appreciate having distance sessions and this mode of delivering treatment is feasible, acceptable and well tolerated (Nelson and Patton, 2016). They are also used for online communication, as some schools have moved to online lessons. Moreover, teenage patients who are able to have remote sessions in their home environment (e.g. their bedroom) may feel more comfortable, thereby facilitating communication with the therapist. However, as adolescents are often less aware of the severity of their eating problem (Dalle Grave and Calugi, 2020), engagement might be more difficult to achieve.

As the complications of malnutrition are more severe in young patients than in adults (Katzman, 2005), it is recommended that these patients are periodically assessed by a physician, both to treat any associated medical complications and to check that their physical state is compatible with distance treatment.

Distance CBT-E with adolescents, as in standard CBT-E (Dalle Grave and Calugi, 2020), includes one 50-minute session with parents alone and several 15–20 minute joint sessions with patients and parents at the end of individual sessions. Both these types of sessions can be conducted online.

In adolescent patients who follow extreme dietary rules that exclude several food groups, or who have lost weight in the last 4 weeks, therapists should provide nutritional education and advice based on National Food and Nutrition Guidelines, adapted for adolescent underweight eating disorder patients, with specific suggestions for meals and snacks using food exchange lists (Dalle Grave and Calugi, 2020). The goal is to devise a flexible plan involving the consumption of foods that are acceptable to the patient and will produce the necessary initial daily energy surplus of 500 kcal. However, as with adults, therapists should be aware that there is a danger that patients may transform this guidance into a dietary rule and take steps to guard against this.

## Guided self-help

In some cases it may not be possible to deliver CBT-E during this time, for example if the patient or therapist does not have sufficient time, enough privacy or suitable technology to follow these guidelines. In this case, for patients who are not underweight, it may be possible to use the guided self-help or pure self-help version of CBT-E, *Overcoming Binge Eating* (Fairburn, 2013), as an alternative.

## Conclusions

The COVID-19 pandemic is creating widespread concern, stress, anxiety and fear, intensified by prolonged social isolation designed to limit the spread of this aggressive disease. Eating disorders are likely to be exacerbated by anxiety about the disease, changes to daily life brought about by the lockdown and more limited opportunities for treatment. Distance CBT-E, a modified form of an evidence-based treatment for all eating disorders, offers a feasible solution to help patients with eating disorders during this difficult period. Despite the challenges of delivering CBT-E by video-calls rather than face-to-face, it is possible to retain most, if not all, aspects of treatment. CBT-E also needs to address some new problems for patients with eating disorders arising from COVID-19. Some of these difficulties may interact with eating disorder psychopathology and usual treatment procedures, rendering treatment more challenging. At the same time, there are potential benefits for some patients during this time and possible opportunities for some aspects of CBT-E to be easier to implement. It is also possible that the introduction and normalising of teletherapy might change the landscape of CBT-E and other therapies in the future. The ability to offer potentially effective treatment through video conferencing may go some way to narrowing the treatment gap by enabling treatment to be available to more of those in need. While it will not meet the scale of the problem, it may encourage further discussion and research about alternative methods of closing the treatment gap for access to psychological treatments (Cooper and Bailey-Straebler, 2015; Kazdin *et al.*, 2017).

## Key practice points

(1) CBT-E is well suited to remote delivery. Many potential challenges of distance CBT-E can be overcome using video-calls, especially where additional features such as screen sharing are also available. Distance CBT-E is not suitable for patients who present a high risk and cannot be managed in this way.
(2) This paper provides guidance on the challenges and potential therapeutic opportunities for delivering CBT-E during the period of the COVID-19 pandemic.
(3) A description of how to adapt CBT-E for remote delivery and to address the additional challenges is provided. This includes modifications to each stage of treatment and adaptations for use with underweight patients and adolescents.
